# A systematic review to evaluate the effectiveness of mental health literacy interventions implemented in schools and communities in low- and middle- income countries

**DOI:** 10.1192/bjo.2021.721

**Published:** 2021-06-18

**Authors:** Niamh McGuckin, Gerard Leavey

**Affiliations:** 1 Ulster University,Maastricht University; 2 Director Bamford Centre for Mental Health & Wellbeing

## Abstract

**Aims:**

Mental health literacy campaigns have received increasing attention as a useful method of reducing the burden of mental disorders, by promoting public awareness and improving attitudes surrounding mental disorders. However, despite the wealth of research into the effectiveness of mental health literacy interventions in high-income countries, there is an absence of evaluations of these interventions in low-middle-income countries (LMICs). This systematic review aims to pool the evidence on effectiveness of these interventions in LMICs.

**Method:**

MEDLINE(OVID), PyschInfo, Scopus and reference lists of included studies were searched. Studies that quantitively measured the effectiveness of mental health literacy interventions amongst schools and communities in LMICs were included, regardless of study design. The included papers were not limited to a particular population demographic, ethnicity or educational level. Studies were included if conducted in LMICs according to the World Bank Classification. Each study was critically assessed according to CASP critical appraisal checklists.

**Result:**

Ten studies met the inclusion and exclusion criteria, including 6 case series, 3 controlled before and after studies and 1 cross sectional study. Most of the studies claimed significant improvement of knowledge, attitudes and coping skills following the intervention. However, the overall the methodological quality of the studies was rated as fair to poor.

**Conclusion:**

The review found that mental health literacy interventions may have promising effects, however the pooled evidence of the effectiveness in LMICs was inconclusive. Further research into the effectiveness of these interventions would benefit from using a RCT design, or controlled-before and after studies, with careful control of confounding variables in order to further establish effect. This study provided insights into the barriers to effective implementation of these programs and examined the contextual appropriateness of such interventions. The review provides recommendations for policy makers for the development of future interventions.

